# Dr. John Braxton Hicks

**Published:** 1897-10

**Authors:** 


### Obituary.

Dr. John Braxton Hicks, F. R. C. P., F. R. S., died at his residence, in Lymington, England, August 28, 1897, aged 72 years. The name of Braxton Hicks has been familiar to the readers of the literature of obstetrics for more than thirty years. He was appointed assistant obstetric physician at Guy's Hospital in 1865, obstetric physician in 1870, and consulting obstetric physician in 1882. He was an honorary fellow of obstetric societies in Great Britain, Germany and the United States. He retired from practice about three years ago. No more appropriate eulogium can be pronounced at this time than that contained in Dr. Wenning's article, published in this edition, in which he frequently alludes to "Braxton Hicks's method."

